# Prognostic Factors and Survival in Pediatric and Adolescent Liposarcoma

**DOI:** 10.1155/2012/870910

**Published:** 2012-09-05

**Authors:** Eric J. Stanelle, Emily R. Christison-Lagay, Emma L. Sidebotham, Samuel Singer, Cristina R. Antonescu, Paul A. Meyers, Michael P. La Quaglia

**Affiliations:** ^1^Pediatric Surgical Service, Department of Surgery, Memorial Sloan-Kettering Cancer Center, 1275 York Avenue, New York, NY 10065, USA; ^2^Gastric and Mixed Tumor Service, Department of Surgery, Memorial Sloan-Kettering Cancer Center, 1275 York Avenue, New York, NY 10065, USA; ^3^Bone and Soft Tissue Pathology Service, Department of Pathology, Memorial Sloan-Kettering Cancer Center, 1275 York Avenue, New York, NY 10065, USA; ^4^Pediatric Oncology Service, Department of Pediatrics, Memorial Sloan-Kettering Cancer Center, 1275 York Avenue, New York, NY 10065, USA

## Abstract

*Purpose*. Liposarcoma is extremely rare in the pediatric population. To identify prognostic factors and determine treatment outcomes, we reviewed our institutional experience with pediatric liposarcoma. *Methods*. We retrospectively reviewed all pediatric patients (age <22 years) with confirmed liposarcoma treated at Memorial Sloan-Kettering Cancer Center. Histologic subtype, tumor location, margin status, recurrence, and adjuvant therapy were analyzed and correlated with overall survival. *Results*. Thirty-four patients (56% male) with a median age of 18.1 years were identified. Twenty-two (65%) had peripheral tumors and 12 (35%) had centrally located tumors. Histologically, 29 (85%) tumors were low grade, and 5 (15%) were high grade pleomorphic. Eleven (32%) had recurrent disease, 9 patients with central tumors and 2 patients with peripheral lesions. Eight deaths occurred, all in patients with central disease. Five-year overall survival was 78%, with a median follow-up time of 5.4 years (range, 0.3–30.3 years). Tumor grade (*P* = .003), histologic subtype (*P* = .01), and primary location (*P* < .001) all correlated with survival, as did stage (*P* < .001) and margin status (*P* = .001). *Conclusions*. Central location of the primary tumor, high tumor grade, and positive surgical margins are strongly correlated with poor survival in pediatric patients with liposarcoma.

## 1. Introduction

Liposarcoma is a nonrhabdomyosarcoma soft tissue sarcoma (NRSTS) arising from primitive mesenchymal cells which undergo lipomatous differentiation. While liposarcomas comprise 20% to 30% of adult NRSTS tumors and account for approximately 2000 new diagnoses in the United States annually, they are much less common in the pediatric population, comprising fewer than 3% of reported cases of NRSTS [1–4]. As previously reported, myxoid liposarcomas show a very characteristic genetic translocation of t(12; 16)(q13; p11), which occurs in greater than 90% of cases. This translocation leads to a fusion of the CHOP and TLS genes, and relevant genetic testing for this characteristic has aided the diagnosis of these rare tumors [5]. Published series of adult patients with liposarcoma have identified tumor grade (low versus high), histologic subtype (well differentiated, dedifferentiated, myxoid, and pleomorphic), and primary tumor location (central versus peripheral) as prognostic variables affecting survival and recurrence [6–13]. In a recently designed nomogram to help predict disease-specific survival (DSS) in adult liposarcoma, investigators at Memorial Sloan-Kettering Cancer Center (MSKCC) incorporated age, histologic variant, tumor burden, primary site, and margins at excision to yield prognostic data for 5- and 12-year survival [12]. However, analyses of pediatric patient populations are needed to guide treatment recommendations and provide insight into the overall prognosis of pediatric liposarcoma. In this study, the largest single-institution review of pediatric patients with liposarcoma, we provide a 20-year update to a previously published report from our institution [14]. 

## 2. Materials and Methods

With Institutional Review Board approval, a retrospective review was conducted to identify patients ≤22 years of age with the pathological diagnosis of liposarcoma who were treated from February 1960 to May 2011. All pathology was re-reviewed by a single pathologist (CRA) with expertise in soft tissue sarcomas. Histologic subtypes were classified according to the World Health Organization classification of tumors of soft tissue and bone. Strict criteria were used to define the histologic subtypes of liposarcomas as well differentiated, dedifferentiated, myxoid/round cell, or pleomorphic, in accordance with previously published recommendations [9, 15, 16]. Pleomorphic and dedifferentiated tumors were considered high grade, while well differentiated and myxoid tumors were classified as low grade. 

Patient medical records were surveyed for sex, age, race, and the nature and duration of presenting symptoms. The primary tumor was classified as central if its location was in the head/neck, thorax (including chest wall), or abdomen/pelvic region. Tumors of either the upper or lower extremities, including both the inguinal and gluteal regions, were considered peripheral. Surgical resection, tumor size, margin status, and forms of adjuvant therapy were also reviewed. Tumor depth was defined as superficial or deep based upon the tumor's position relative to the investing fascia of the extremity, chest, or abdominal wall. All intra-abdominal or intrathoracic tumors were considered deep. The overall stage of disease was determined based upon the tumor-node-metastasis (TNM) classification and was consistent with the American Joint Committee on Cancer (AJCC) staging system for soft tissue sarcoma [17].

Statistical analyses were performed using SPSS software version 19.0 for Windows (SPSS Inc., Chicago, IL). Descriptive statistics were used for all patient characteristics, and overall survival (OS) and progression-free survival (PFS) were evaluated using the Kaplan-Meier method. OS calculations were based on the time from the date of diagnosis to either the date of confirmed death or the date of last recorded contact with the patient. PFS was calculated based on the time from diagnosis to the date of disease progression or relapse. The log-rank test was implemented to compare survival between groups, and the chi-square test was used to compare categorical data. Statistical significance was defined as *P* < .05. 

## 3. Results

### 3.1. Patient Demographics

Thirty-four patients with a pathological diagnosis of liposarcoma were identified. Nineteen patients were male (56%) and 15 were female (44%). The median age at diagnosis was 18.1 years (range, 0.3–22.8 years). The age distribution was as follows: 1 patient aged 0–5 years, 1 patient aged 6–10 years, 7 patients ages 11–15 years, 21 patients ages 16–20 years, and 4 patients ages 21-22 years. Patients presented after a mean of 3 months (range, 0–60 months) of symptoms. Patients most commonly presented with an enlarging mass (*n* = 28) or pain and abdominal distention (*n* = 3). Two patients were asymptomatic at presentation with a mass that was incidentally discovered by imaging for unrelated causes. Presenting symptoms were unspecified in one patient. Twenty-eight patients were white, 2 were black, 3 were Hispanic/Latino, and 1 patient was Asian. 

A majority of patients (*n* = 22; 65%) had peripheral lesions, with tumor sites in the lower extremities (*n* = 16), inguinoscrotal region (*n* = 5), and upper limb (*n* = 1). Twelve patients had central tumors, located in the head/neck (*n* = 2), the paraspinal/posterior mediastinal region (*n* = 2), the chest wall/shoulder girdle (*n* = 3), and the retroperitoneum/abdomen/pelvis (*n* = 5). Median tumor size was 6.0 cm (range, 2–21 cm). The mean size of central tumors at presentation (11.5 cm (range, 4–21 cm)) was nearly twice that of peripheral tumors (5.9 cm, (range, 2–16 cm); *P* = .01). Twenty (59%) of the 34 patients in our cohort had deep lesions, with 11 tumors (55%) located peripherally and 9 (45%) centrally. Deep lesions (mean size, 10.4 cm) were larger than superficial lesions (mean size, 4.3 cm) (*P* < .001).

Twenty-nine patients (85%) had low-grade tumors, among which myxoid histology was the most common subtype (25 of 29; 86%). The remaining low-grade tumors were well differentiated (*n* = 4; 14%). All 5 high-grade tumors displayed pleomorphic histology (5 of 34; 15%), and 4 of these were centrally located. Although unusual subtypes such as spindle cell myxoid and pleomorphic myxoid can sometimes occur, as described recently [18], no patients in our series met those criteria, based on our formal pathological re-review. Two patients presented with metastatic disease. Both had centrally located, high-grade pleomorphic tumors. Despite multiple resections and adjuvant treatment with both aggressive chemotherapy and external-beam radiation therapy (EBRT), both of these patients eventually succumbed to disease progression. 

### 3.2. Treatment

All patients underwent surgical resection of the primary tumor (Table 1). This was the sole treatment for 19 (56%) patients. Among those 19 cases, 3 (16%) had positive surgical margins, 2 with peripheral lesions (both in the lower extremity and both with myxoid histology) and 1 with a central tumor (retroperitoneal tumor with well-differentiated histology). Seven (21%) of 34 patients received adjuvant radiation therapy only (EBRT in 6, and intraoperative radiation therapy (IORT) in 1), 2 of 34 patients received chemotherapy only, and 6 (18%) of 34 patients underwent both chemotherapy and radiation. Radiation doses ranged from 2500 to 8400 cGy, with a mean dose of 5400 cGy. Dosage could not be confirmed for 2 patients. No patients received neoadjuvant therapy. Ten (83%) of the 12 patients with centrally located tumors received some form of adjuvant therapy while only 5 (23%) of the 22 patients with peripheral tumors had additional therapy.

Surgical margins after the first definitive procedure were positive in 12 (35%) patients and negative in 22 (65%). Four (33%) patients with positive margins, all with central tumors, had gross residual disease (R2 resection) at the completion of surgery. Overall, 75% (9/12) of patients with positive margins had central tumors. 

### 3.3. Outcomes

**Figure 1:**
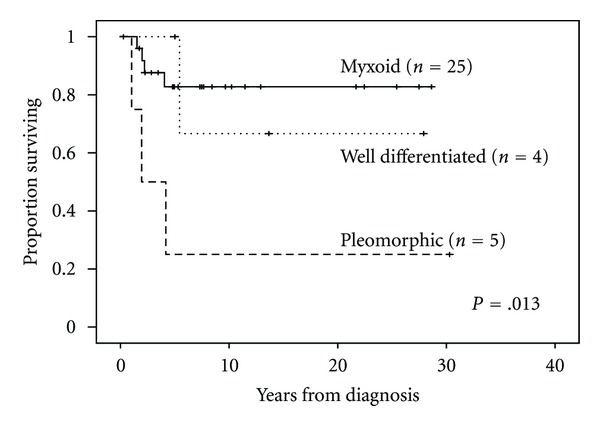
Overall survival for different subtypes of liposarcoma.

**Figure 2:**
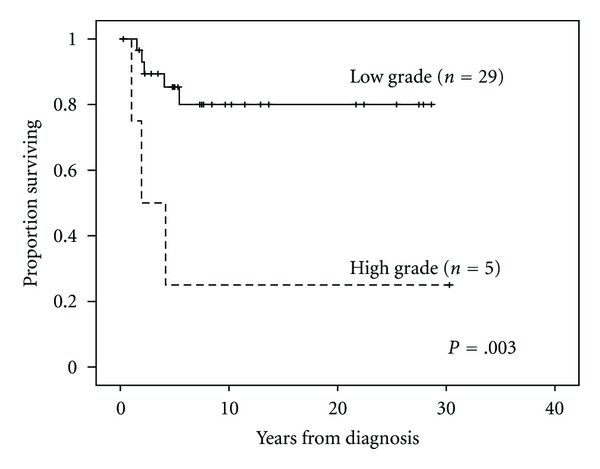
Overall survival and tumor grade.

**Figure 3:**
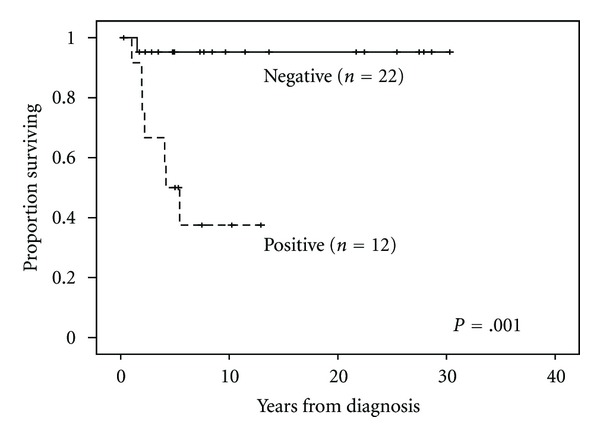
Overall survival and margin status.

The median followup for the entire cohort was 5.4 years (range, 0.3–30.3 years). There was no difference in survival between male and female patients. Tumor grade and histology correlated most significantly with survival. Patients with high-grade pleomorphic tumors had a median survival of 1.9 years (range, 0.3–30.3 years) versus 7.3 years (range, 1.5–28.6 years) in those with low-grade myxoid/well-differentiated tumors (*P* = .003). When comparing histologic subtype and OS, patients with myxoid, well-differentiated, and pleomorphic histologies had 5-year estimated survival rates of 83%, 67%, and 25%, respectively (*P* = .01) (Figures 1 and 2). Median followup among patients with negative margins (8.0 years (range, 0.3–30.3 years); *n* = 22) was significantly longer than for those with positive margins (4.6 years (range, 1.0–12.9); *n* = 12; *P* = .001). Five-year OS for patients with negative margins was 95% compared to 50% for patients with positive margins (*P* = .001) (Figure 3). See Table 2 for univariate analysis of prognostic factors and survival.

**Figure 4:**
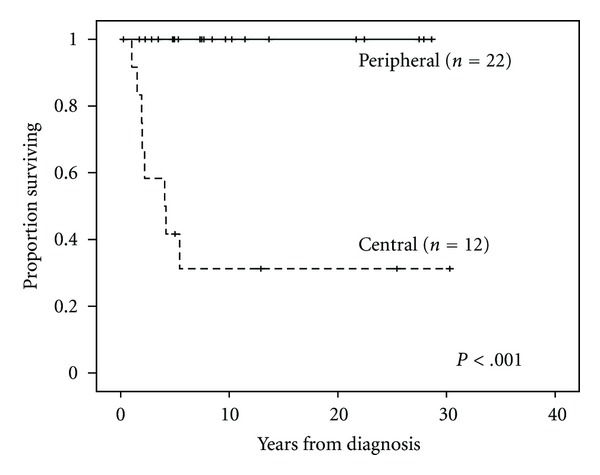
Overall survival for peripheral versus central primary tumor location.

There were 8 (24%) deaths in this series, all from progression of disease, and all in patients with centrally located lesions. In contrast, all patients with peripheral lesions are alive and well. The Kaplan-Meier analysis demonstrated the significant correlation of central lesion location with poorer survival (*P* < .001) (Figure 4). Eleven patients (31%) experienced recurrence of disease, which correlated with shorter OS (*P* < .001). Recurrence was more likely in central (9 of 12; 75%) than in peripheral (2 of 22; 9%) tumors (*P* < .001). PFS was not significantly different when comparing central and peripheral lesions (*P* = 0.64). Of the 11 patients with recurrent disease, 3 (27%) had high-grade pleomorphic tumors, 7 (64%) had low-grade myxoid-type lesions, and 1 (9%) had a low-grade well-differentiated tumor. Margin status and recurrence of disease were significantly correlated. Disease recurred in 8 (67%) of 12 patients with positive margins, compared to 3 (14%) of 22 patients with negative margins (*P* = .002). Two cases of recurrent disease were associated with primary tumors in the periphery, both in the lower extremity and both with initially negative margins. Tumor size (≤ or >5 cm) did not affect OS (*P* = 0.3) or the rate of recurrence (*P* = 0.7). In the 15 (43%) patients that received adjuvant therapy (7 radiation only, 2 chemotherapy only, and 6 with both), median OS was 4.2 years (range, 0.3–27.9 years). For patients who did not receive adjuvant chemo- and/or radiotherapy, median survival was 7.5 years (range, 1.7–28.6 years; *P* < .001). All 8 patients who succumbed to their disease had received adjuvant therapy and, based on serial imaging, there was no evidence that administration of chemotherapy slowed disease progression. Six (75%) of these 8 patients received both adjuvant chemotherapy and radiation therapy, while one had only chemotherapy, and the remaining patient had only radiotherapy. The 2 patients that presented with metastatic disease both died secondary to progression, despite aggressive surgical and adjuvant chemotherapy/radiation therapy. These patients had survival times of 1.0 and 4.2 years.

Two patients with high-grade, centrally located tumors presented with metastatic disease in the lung and/or bone. Three additional patients, all with central tumors, developed lung and/or bone metastases during the course of followup. Of the 5 patients with metastatic disease, 4 have died due to disease progression. The fifth was a foreign national lost to followup, but is presumed to have died of disease.

## 4. Discussion

Nonrhabdomyosarcoma soft tissue sarcomas, though rare, comprise a heterogeneous group of tumors that respond differently to chemo- and radiotherapy; have vastly different local, locoregional, and distant metastatic potential; and are associated with disparate overall survival rates. Their rarity and heterogeneity have made it difficult to gather prospective data on sufficient numbers of patients to be able to predict disease progression and to generate optimized paradigms of care. Because liposarcoma is among the most common types of NRSTS in adults, several large retrospective series analyzed during the past decade (which in aggregate account for more than 1500 cases) have provided a foundation for understanding the presentation and progression of adult liposarcoma and its treatment response [6–9, 16]. 

In contradistinction to NRSTS in adults, liposarcoma in the pediatric population is very rare. Accordingly, there are very few published studies of any size that attempt to correlate pediatric disease presentation and tumor biology with outcome or to differentiate the presentation and progression in children from that of adults. Moreover, the largest of these studies have been multi-institutional and have spanned the course of decades, further complicating the synthesis and analysis of data. We present here the largest series of patients with pediatric liposarcoma. Our results suggest that the presentation of pediatric liposarcoma is different from its adult counterpart and that the two most significant factors that affect overall survival are the location of the primary tumor and the completeness of surgical excision. As observed in other studies, we found that pediatric patients are more likely to present with histologically low-grade tumors (29 of 34; 85%) than adults and, within this category, are more likely to present with myxoid histology (25 of 29; 86%) than adults, in whom well-differentiated histology predominates [18–20]. Unlike reports of adult liposarcoma dedifferentiation into higher-grade tumors over time [8, 12], our current series did not reveal any dedifferentiated tumors. Only 15% of patients demonstrated high-grade (pleomorphic) histology, less than half the percentage typically reported in adult studies; however, 80% of these were centrally located. Multivariate analysis would be helpful in elucidating which factor has the greatest impact on survival; unfortunately, due to the rare nature of pediatric LS, even this relatively large review does not have a sufficient number of patients to adequately power multivariate statistics. 

Surgical excision was the mainstay of treatment for all patients in this cohort. Only two cases of recurrent disease were associated with primary tumors in the periphery, both in the lower extremity and both with initially negative margins, a rate of local recurrence (2 of 22; 9%) similar to that noted in adult studies of peripheral tumors with complete excision [6]. Both of these patients had low-grade, myxoid-type tumors in the proximal thigh and neither of these patients received adjuvant therapy. After recurrence, wide surgical reexcision was conducted (both with negative margins) and neither patient received any adjuvant therapy. Currently, both are alive and well with no evidence of disease. Although this current study is too small to make definitive recommendations about the use of adjuvant therapy, surgical excision with a negative margin may be sufficient treatment for low-grade tumors in peripheral locations. It may also be feasible to treat local recurrence of low-grade tumors of the extremities with reexcision alone. Our cohort only had one patient with a high-grade pleomorphic tumor in the periphery. This patient initially underwent surgery with negative margins and then received multiple cycles of doxorubicin and ifosfamide. No radiation was offered, and this patient currently has no evidence of disease. Three patients with low-grade peripheral tumors had positive microscopic margins after surgery; none received any additional surgery, but one of these patients received adjuvant radiotherapy. All are alive with no evidence of disease. Nonetheless, surgical therapy alone without reexcision for positive microscopic margins is controversial. Most practitioners recommend reexcision or adjuvant therapy for microscopic residual disease in patients with liposarcoma; however, current Children's Oncology Group studies are attempting to evaluate whether avoiding adjuvant treatment after marginal resection of a low-grade tumor is safe [6, 19, 21, 22]. 

Central tumors have a much poorer prognosis than peripheral lesions, with a 5-year survival of only 43%. This poorer prognosis is likely due to a combination of later presentation and more difficulty in achieving a complete resection. Central lesions may also have more aggressive tumor biology, as observed in this study, in which 4 of the 5 pleomorphic tumors were centrally located. Three of 4 patients with central pleomorphic tumors died of disease; 2 of these experienced disease recurrence less than a year after primary resection. Among the 8 patients who died of disease, all had centrally located tumors, and all but one had positive surgical margins after the first definitive resection. Only 2 patients with centrally located tumors did not receive adjuvant therapy. Of these, one had a high-grade pleomorphic tumor of the chest wall/shoulder girdle with negative margins after surgery. The other patient had a low-grade tumor in the retroperitoneum. Both are alive with no evidence of disease. 

The role of chemotherapy is poorly defined in pediatric patients with LS. In our series, chemotherapy was administered primarily to patients with central tumors. The only patient with a peripheral lesion who received chemotherapy had a high-grade pleomorphic tumor of the right buttock that exhibited rapid enlargement during the 2 months prior to excision. Diagnosis of liposarcoma was commonly delayed in patients with central tumors, in whom larger tumor size and high-grade histology were also associated with a higher rate of positive surgical margins. In this population of patients with advanced disease, adjuvant therapy was offered as a salvage technique in the hope of extending survival, and thus it was no surprise that adjuvant therapy was associated with decreased survival when compared to those patients who did not receive additional therapies. However, size limitations in this study precluded meaningful statistical analysis of the contribution of adjuvant therapy to progression-free or overall survival. Some adult studies [7, 13], as well as several pediatric reports [19, 22], have suggested a potential role for neoadjuvant therapy to aid in the cytoreduction of intra-abdominal and retroperitoneal tumors prior to operative excision, but the exact role of neoadjuvant therapy has yet to be defined. 

Although this study is the largest single-institution report of pediatric liposarcomas, the strength of our analysis is limited by its retrospective nature, relatively small sample size, and 50-year study period (during which time significant changes in the diagnosis and treatment of soft tissue sarcomas have occurred). We are optimistic that the NRSTS database currently being compiled by the Children's Oncology Group will provide more comprehensive insight into the presentation and progression of pediatric liposarcoma, as well as its response to treatment. 

In conclusion, pediatric liposarcoma appears to have a presentation and clinical course that is distinct from its adult counterpart. A majority of tumors are peripherally located with myxoid histology; patients with these tumors have an excellent overall prognosis following surgical excision with negative margins as single-modality treatment. High-grade tumors are rare, but are associated with a more aggressive course and poorer overall survival. Optimized treatment algorithms with neoadjuvant and/or adjuvant therapies have yet to be defined. Patients with centrally located tumors have a poor prognosis, due in part to a greater percentage of high-grade histologies, and the difficulty in achieving negative resection margins in such lesions. These are the patients who would most benefit from multicenter, randomized trials evaluating the role of neoadjuvant and adjuvant therapies.

## Figures and Tables

**Table 1:** Treatment and outcomes for 3 different histologic tumor subtypes.

	Myxoid (*n* = 25)	Well differentiated (*n* = 4)	Pleomorphic (*n* = 5)	*P* value
Treatment				
Surgery	16	2	1	.08
Surgery + XRT	6	1	0	
Surgery + XRT + CT	2	1	3	
Surgery + CT	1	0	1	

Median PFS (years)	0.3	3.1	0.5	.34

Mean radiation dose, Gy (range)	55 (42–67)	35 (24–45)	64 (50–84)	.38
Median followup (years)	7.3	5.4	1.9	.01
Number of recurrences	7	1	3	.31

Median followup (years) for patients with recurrence	8.38	00	2.39	<.001

XRT: radiation therapy; CT: chemotherapy; PFS: progression-free survival.

**Table 2:** Univariate analysis evaluating 5-year survival.

	*n* (%)	Number of events	5-Year survival	95% CI	*P* value
Overall survival	34 (100%)	8	78%	63–93	

Male	19 (56%)	4	76%	55.2–96.8	.71
Female	15 (44%)	4	79%	57–100	

High grade	5 (15%)	3	25%	0–68.4	.003
Low grade	29 (85%)	5	85%	71.4–98.6	

Well differentiated	4 (11%)	1	100%	0	.013
Myxoid	25 (74%)	4	83%	67.2–98.8	
Pleomorphic	5 (15%)	3	25%	0–68.4	
Dedifferentiated	0		0%		

Tumor ≤ 5 cm	14 (41%)	2	84%	63.2–100	.312
Tumor > 5 cm	20 (59%)	6	74%	53.6–94.4	

(+) Margins	12 (35%)	7	50%	21.2–78.8	.001
(−) Margins	22 (65%)	1	95%	85.8–100	

Peripheral	22 (65%)	0	100%	0	<.001
Central	12 (35%)	8	42%	13.6–70.4	

Superficial	14 (41%)	1	92%	79.2–100	.103
Deep	20 (59%)	7	69%	47.6–90.4	

Stage 1A	11 (32%)	1	91%	73.5–100	<.001
Stage 1B	16 (47%)	2	86%	67.3–100	
Stage 2A	2 (6%)	0	100%	0	
Stage 2B	0	0	0%	0	
Stage 3	3 (9%)	3	33%	0–87.7	
Stage 4	2 (6%)	2	NR	0	
